# Long-Chain Bases from Sea Cucumber Alleviate Obesity by Modulating Gut Microbiota

**DOI:** 10.3390/md17080455

**Published:** 2019-08-01

**Authors:** Shiwei Hu, Yangli Xu, Xiang Gao, Shijie Li, Wei Jiang, Yu Liu, Laijin Su, Huicheng Yang

**Affiliations:** 1 Innovation Application Institute, Zhejiang Ocean University, Zhoushan 316022, China; 2 Wenzhou Academy of Agricultural Sciences, Wenzhou 325006, China; 3 College of Life Sciences, Qingdao University, Qingdao 266071, China; 4 Zhejiang Marine Development Research Institute, Zhoushan 316021, China

**Keywords:** sea cucumber, long-chain bases, obesity, gut microbiota, lipopolysaccharide, short chain fatty acids

## Abstract

This study evaluated the effects of long-chain bases from sea cucumber (SC-LCBs) on modulation of the gut microbiota and inhibition of obesity in high fat diet-fed mice. Results showed that SC-LCBs exerted significant antiobese effects, which were associated with the inhibition of hyperglycemia and lipid accumulation. SC-LCBs also regulated serum adipocytokines toward to normal levels. SC-LCBs caused significant decreases in Firmicutes, Actinobacteria phylum, and obesity-related bacteria (*Desulfovibro*, *Bifidobacterium*, *Romboutsia* etc. genus). SC-LCBs also elevated Bacteroidetes, Proteobacteria, Verrucomicrobia phylum, and short chain fatty acids (SCFAs)-producing bacteria (*Bacteroides*, *Lactobacillus*, *Lachnospiraceae_NK4A136_group* etc. genus). Moreover, serum and fecal lipoplysaccharide (LPS) concentrations and its dependent toll-line receptor 4 pathway were inhibited by SC-LCBs treatment. SC-LCBs caused increases in fecal SCFAs and their mediated G-protein-coupled receptors proteins. These suggest that SC-LCBs alleviate obesity by altering gut microbiota. Thus, it sought to indicate that SC-LCBs can be developed as food supplement for the obesity control and the human gut health.

## 1. Introduction

Excess accumulation of body fat mass resulting from unhealthy dietary patterns and lifestyles directly induces obesity. The prevention of obesity has remained a huge challenge for modern researchers because of its high prevalence [1]. The high incidence of obesity leads to a series of public health problems and increases the risks of various diseases, including type 2 diabetes, fatty liver, hyperlipemia, atherosclerosis, cardiovascular disease, and even cancer [2]. However, among the complex factors affecting the progression of obesity, the gut microbiota plays an important role in host nutrient digestion and energy metabolism [3]. Accumulating data support the idea that the gut microbiota represents a novel way for the control of obesity and its related diseases [4,5,6]. Particularly, the increase in Firmicutes, and the reductions in Bacteroidetes, *Desulfovibro*, and *Bifidobacterium* (obesity-related bacteria) positively lead to the development of obesity in human and rodents [7,8], but such clinical studies reported that there may be no significant association between obesity and the abundances of Firmicutes and Bacteroidetes [9,10], implying that obesity can also be affected by other factors. In addition, obese animals tend to have a higher lipopolysaccharide (LPS) level and lower short chain fatty acids (SCFAs) concentrations [11]. Gram-negative bacteria-derived LPS has been defined as the primum movens in the development of obesity and its related metabolic diseases through binding with toll-line receptor 4 (TLR4) and subsequently activating CD14 [12]. Moreover, acetate, propionate, and butyrate produced by specific beneficial bacteria, participate in the well balanced control of opposing lipolysis and lipogenesis by triggering their receptors, G protein-coupled receptors (GPRs) [13,14]. These imply that modulation of gut microbiota may be an optimal strategy for controlling obesity and its associated metabolic syndrome.

Sea cucumber, which has received intensive attention in recent years, is a traditional food and medicine in China and other Asian countries, and rich in various bioactive substances, including several lipids, polysaccharides, saponins, etc. Prior studies have reported that only polysaccharides were proved to modulate gut microbiota. Sulfated polysaccharide and its depolymerized derivative from sea cucumber can prevent obesity via modification of gut microbiota in high fat diet (HFD) mice [15]. This polysaccharide also modulated the gut microbiota and its metabolites in normal mice [16]. Fucoidan, a kind of polysaccharide from the sea cucumber *Pearsonothuria graeffei*, could alleviate gut microbiota dysbiosis and metabolic syndromes in HFD-fed mice [17]. Another polysaccharide, fucosylated chondroitin sulphate from sea cucumber, was reported to exhibit anti-inflammatory effects by altering gut microbiota in obese mice [18]. However, other bioactive substances on influencing gut microbiota have not been involved in any paper. Belonging to the family glycosphingolipids, also named sphingoid bases, is the simplest members. Increasing interest has been focused on LCBs from marine organisms, especially from sea cucumber (SC-LCBs) [19]. It was reported that SC-LCBs could induce apoptosis in human hepatoma HepG2 cells [20] and inhibit HepG2 cell proliferation [21]. SC-LCBs also protected against HFD-induced metabolic disorders [22] and endoplasmic reticulum stress-associated inflammation in mice [23]. Our previous study proved that SC-LCBs could inhibit adipogenesis and regulated lipid metabolism in 3T3-L1 adipocytes [24].

Since SC-LCBs mitigated metabolic disorder, inflammatory response, and adipogenesis, we can speculate that the modulation of gut microbiota may be involved in these processes. Therefore, the present study was conducted to evaluate how SC-LCBs alleviate obesity and modulate gut microbiota. In addition, the mechanism by which SC-LCBs regulated the secondary metabolites of gut bacteria and their dependent signal transduction was also investigated.

## 2. Results

### 2.1. SC-LCBs Alleviated Obesity

**Figure 1:**
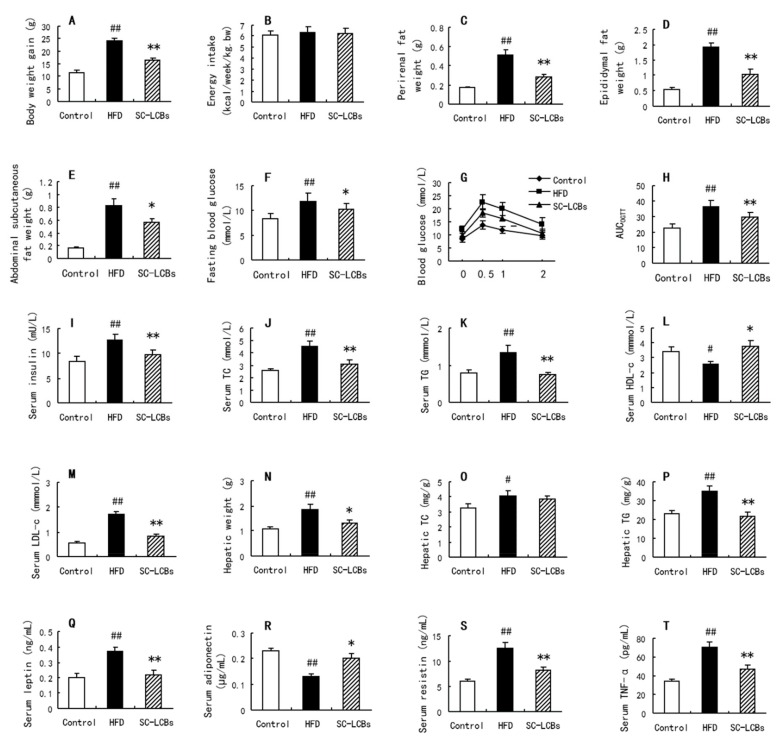
Effects of SC-LCBs on body and serum parameters in HFD-fed mice (*n* = 10). (**A**), body weight gain; (**B**), energy intake; (**C**), perirenal fat weight; (**D**), epididymal fat weight; (**E**), abdominal subcutaneous fat weight; (**F**), fasting blood glucose; (**G**), blood glucose in OGTT; (**H**), AUC in OGTT; (**I**), serum insulin; (**J**), serum TC; (**K**), serum TG; (**L**), serum HDL-c; M, serum LDL-c; (**N**), hepatic weight; (**O**), hepatic TC; (**P**), hepatic TG; (**Q**), serum leptin; (**R**), serum adiponectin; (**S**), serum resistin; (**T**), serum TNF-α. ^##^ *p* < 0.01 vs. control mice; * *p* < 0.05, ** *p* < 0.01 vs. HFD mice.

**Figure 2:**
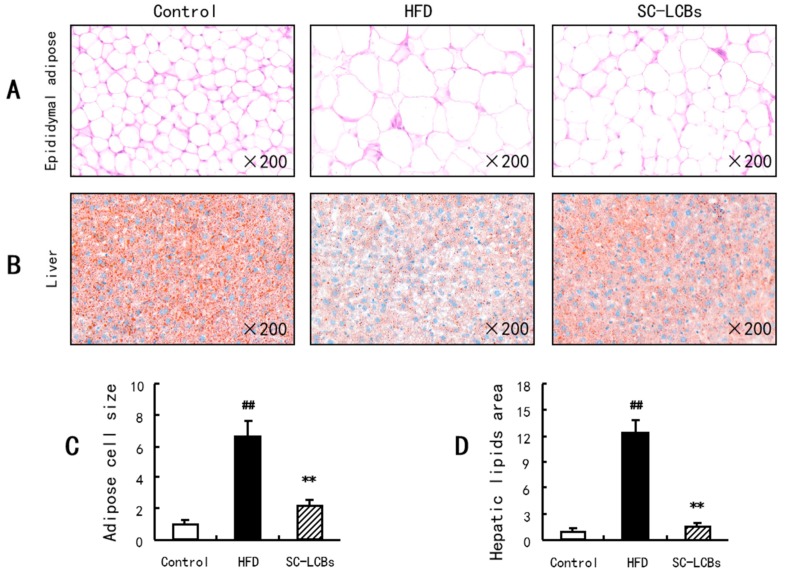
Effects of SC-LCBs on the histology of adipose and liver tissues using H&E staining (×200, *n* = 10). The adipose cell size and hepatic lipids area were measured by CaseViewer 2.0, and the size or area of the control was defined as 1. (**A**), H&E staining to epididymal adipose; (**B**), H&E staining to liver; (**C**), adipose cell size, the bars means the quantized values for each groups in (**A**); (**D**), hepatic lipids area, the bars means the quantized values for each groups in B. The difference between the control and HFD mice, between HFD and SC-LCBs groups was analyzed using Student’s test. ^##^ *P* < 0.01 vs. control; ** *P* < 0.01 vs. HFD.

As shown in Figure 1A, HFD-induced elevation in body weight was decreased by 28.80% when the mice were administrated with SC-LCBs. However, there were no significant changes in energy intake among the three groups, suggesting that the animals in this experiment were under similar calorie circumstances. Figure 1C,D and E show that the weights of perirenal fat, epididymal fat, and abdominal subcutaneous fat were all significantly reduced by 45.53%, 46.35%, and 27.63% in SC-LCBs group compared with HFD group, respectively (*p* <  0.05, *p* <  0.01). Moreover, HFD induced a remarkable increase in epididymal adipose size, while SC-LCBs feeding dramatically inhibited the increase by 66.09% (*p  *<  0.01) (Figure 2). These data indicate that SC-LCBs can alleviate HFD-induced obesity by decreasing adipose cell size and fat mass.

### 2.2. SC-LCBs Inhibited Hyperglycemia

Hyperglycemia occurs in the process of HFD-induced obesity. When treated with SC-LCBs, the mice showed 10.29% and 22.38% reductions in fasting blood glucose and serum insulin levels compared with obese animals, respectively (Figure 1F,I) (*p * <  0.05, *p * <  0.01). In oral glucose tolerance test (OGTT) experiment, SC-LCBs also improved the impaired glucose tolerance (Figure 1G,H). These indicate that SC-LCBs-improved hyperglycemia is relative to the anti-obese effects.

### 2.3. SC-LCBs Reduced Lipids Accumulation

Hyperlipemia is a characteristic disorder in obese individuals. As shown in Figure 1J,K,M,N, HFD caused significant increases in serum TC, TG and LDL-c levels (*p * <  0.05, *p * <  0.01) and decreases in serum HDL-c level (*p * <  0.01). SC-LCBs-treated mice displayed obvious reductions in serum TC and TG by 32.68% and 45.19%, respectively. Moreover, serum LDL-c was decreased by 53.18% in SC-LCBs treated mice compared with HFD animals, while serum HDL-c was elevated by 45.53%. These indicate that SC-LCBs inhibit obesity-induced hyperlipemia.

In obese individuals, lipid accumulation occurs in peripheral tissues, including adipose tissue, liver tissue, etc. In the present study, hepatic weight in obese mice was higher than that in control group (Figure 1N), which is associated with the remarkable increase in the hepatic lipids amount (Figure 2). When treated with SC-LCBs, the HFD-fed mice showed significant reductions in hepatic weight and lipids area (*p * <  0.05, *p * <  0.01). Furthermore, SC-LCBs also caused a significant decrease in TG concentration in the liver of obese mice (*p  *<  0.01), through there was no significant change in hepatic TC level between HFD group and SC-LCBs group. These suggest that SC-LCBs can mitigate hepatic lipid accumulation.

### 2.4. SC-LCBs Regulated Serum Adipokines

Adipokines play an important role in the development of obesity. The obese mice treated with SC-LCBs showed significant decreases in serum resistin, leptin, and tumor necrosis factor-α (TNF-α) concentrations (*p  *<  0.01), and obvious increase in serum adiponectin concentration (*p  *<  0.05). These suggest that SC-LCBs can regulate circulatory adipokines in obese mice.

### 2.5. SC-LCBs Modulated Gut Microbiota

**Figure 3:**
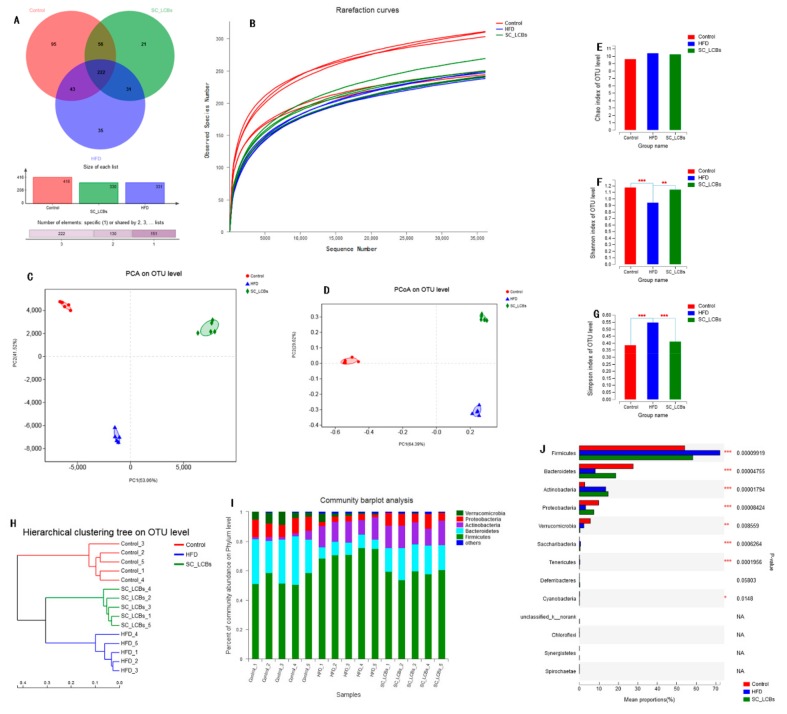
Effects of SC-LCBs on the composition of the gut microbiota in HFD mice (*n* = 5). (**A**), Vean diagram; (**B**), rarefaction curves; (**C**), PCA on OTU level; (**D**), PCoA on OTU level; (**E**), chao index; (**F**), shannon index; (**G**), simpon index; (**H**), hierarchical clustering tree on OTU level; (**I**), community barplot analysis fro each sample; J, bacteria at phylum level for each group. Univariate differential abundance of OTUs at the Phylum level was tested by incorporating Fisher’s exact test and the false discovery rate (FDR) among control, HFD, and SC-LCBs groups and between mouse genotypes. *P* values were corrected with the Benjamini-Hochberg method to correct for the false discovery rate across multiple comparisons, which were generated using Metastats and considered significance at *p  *<  0.05. The difference between the control and HFD mice, between HFD and SC-LCBs groups was analyzed using Student’s test. * *p* < 0.05, ** *p* < 0.01 *** *p* < 0.001 vs. HFD.

Obesity could be developed and exacerbated by the intestinal bacteria dysbiosis in the host. To investigate the protective activities of SC-LCBs on maintenance of microbial community in obese mice, the V3-V4 of 16S rRNA gene from the fecal samples was carried out. The Venn diagram data analysis showed that each group had their own distinct operational taxonomic units (OTUs, Figure 3A). The number of sequences number plateaued at 37,000 (Figure 3B), suggesting that the sequencing depth achieved was sufficient for this study. Alpha diversity was used to express the within-community richness and diversity. Figure 3E showed Chao index (reflecting richness), while Figure 3F,G showed Shannon and Simpson indexes (reflecting the community diversity). There was no significant difference in Chao index between the control, HFD, and SC-LCBs groups, suggesting that the species richness of the bacteria in C57BL/6J mice was not affected by HFD feeding or SC-LCBs treatment. Significant increases in Shannon index and decreases in Simpson index were observed in SC-LCBs group compared with HFD group, implying that the diversity of the gut microbiota was remarkably increased by SC-LCBs. The relationship between the community structures of the three groups was assessed using the Principal Component Analysis (PCA) and Principal Coordinates Analysis (PCoA) methods on the OUTs levels (Figure 3C,D). The data showed that the gut microbiota was distinct from each group, and the Hierarchical clustering tree on OUTs levels was also showed distinct separation of the microbial composition (Figure 3H). Collectively, these results indicate that the gut microbiota in obese mice was modulated by SC-LCBs.

**Figure 4:**
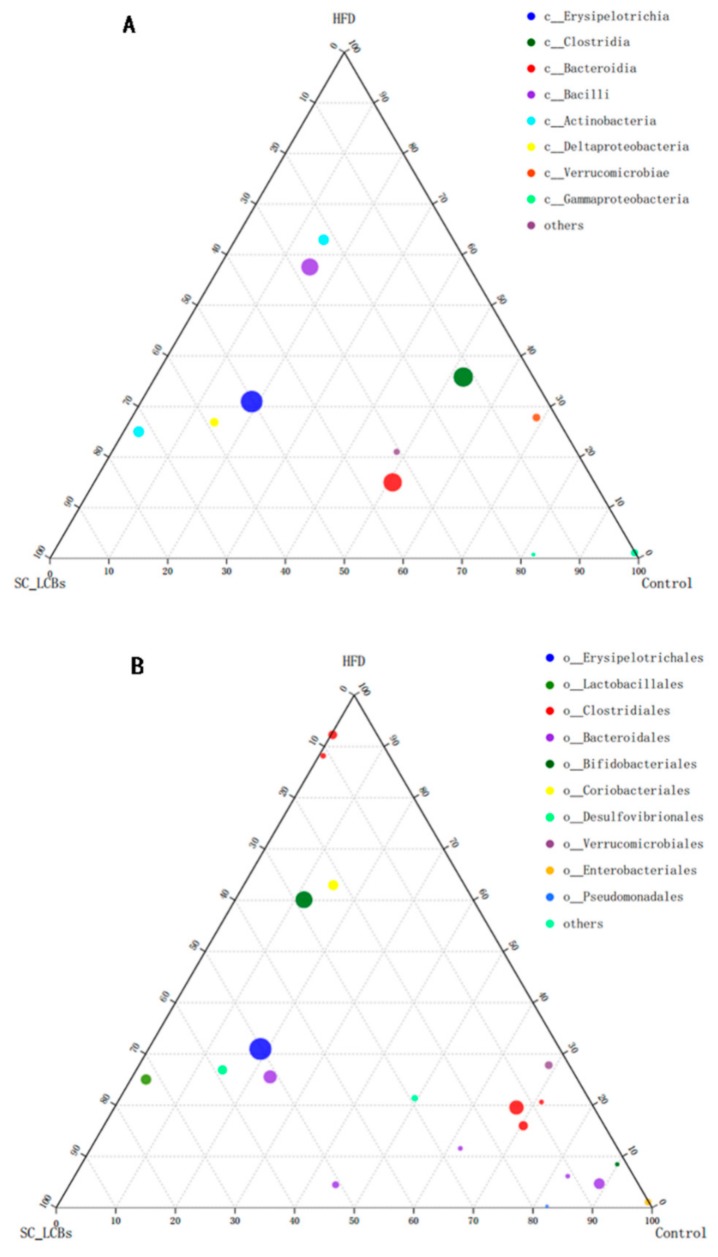
Effects of SC-LCBs on gut microbiota at class and order levels using ternaryplot method (*n* = 5). (**A**), gut microbiota at class level; (**B**), gut microbiota at order level.

The microbial composition of the three groups was further analyzed at various taxonomic levels. At the phylum level, HFD induced obvious increases in the abundances of Firmicutes and Actinobacteria, but decreases in Bacteroidetes, Proteobacteria, and Verrucomicrobia (Figure 3I,J). SC-LCBs treatment was found to considerably modify the relative abundances of Firmicutes, Bacteroidetes, and Proteobacteria, similar to the control group. The changes of gut microbiota at order and class levels using a ternary plot method are shown in Figure 4. 

Compared with the HFD group, SC-LCBs remarkably enhanced the abundances of *Erysipelotrichia*, *Bacteroidia*, *Deltaproteobacteria*, and *Verrucomicrobiae*, and reduced *Clostrida*, *Bacilli*, and *Actinobacteria* at class level. SC-LCBs-treated mice also showed significant increases in *Erysipelotrichales*, *Bacteroidales*, *Lactobacillales*, and *Desulfovibrionales*, and decreases in *Bifidobacteriales* and *Coribacteriales* at order level. 

**Figure 5:**
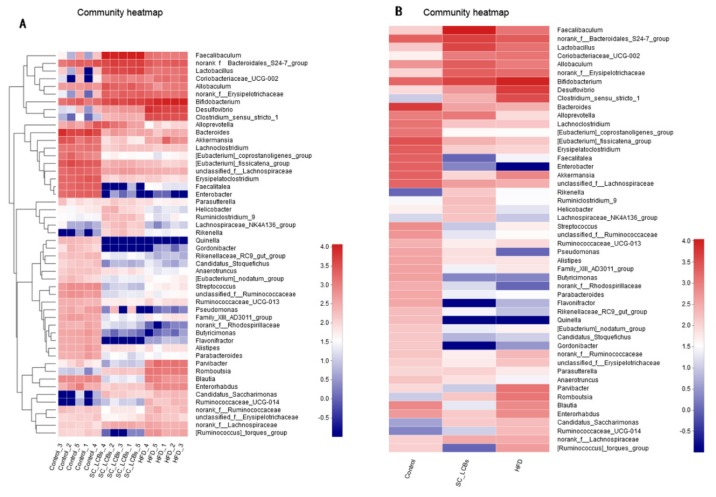
Response of gut microbiota at the genus level to SC-LCBs treatment (*n* = 5). The heatmap is colour-coded based on row Z-scores. (**A**), heatmap indicating relative contribution of the top 50 dominat genera in each sample; (**B**), heatmap in each group.

Next, to determine which bacterial genus shaped the distinct microbiota structures between three groups, the relative abundances of bacteria genus were tabulated on a heat map (Figure 5). There were 44 genuses with significant difference between control group and HFD group, while 41 genuses differed between the HFD group and SC-LCBs group, implying that the protective effects of SC-LCBs on mouse obesity may be mediated by a subset of the bacterial taxa. The numbers of *unclassified_f__Ruminococcaceae*, *Ruminococcaceae_UCG-013*, *norank_f__Ruminococcaceae*, *unclassified_f__Erysipeloterichaceae*, *Ruminococcaceae_UCG-014*, and *[Ruminococcus]_torques_group*, belonging to Firmicutes, were lower in SC-LCBs-treated mice compared with the HFD group, while *norank_f__Erysipelotrichaceae* was increased. Moreover, other genus bacteria, which are positively correlated with obesity, were also decreased in SC-LCBs-treated obese mice, such as *Coriobacteriaceae_UCG-002*, *Desulfovibro*, *Bifidobacterium*, *Parvibacter*, *Romboutsia*, and *Enterorhabdus*. Notably, the abundances of the SCFAs-producing microbiota, including *Faecalibaculum*, *norank_f_Bacteroidales_S24-7_group*, *Lactobacillus*, *Allbaculum*, *Bacteroides*, *Alloprevotella*, *unclassified_f__Lachnospiraceae*, *Lachnospiraceae_NK4A136_group*, *Rikenella*, and *Ruminiclostridium_9* were increased in SC-LCBs-supplemented mice. In addition, *Rikenellaceae_RC9_gut_group* and *Parasutterella* (belonging to Bacteroidetes) were enriched by SC-LCBs treatment in obese mice.

### 2.6. SC-LCBs Regulated Secondary Metabolites of Gut Microbiota

The gut microbiota was modulated by SC-LCBs supplementations in HFD-fed mice, thus, we next investigated their effects on microbial metabolites: LPS and SCFAs. As shown in Table 1, SC-LCBs treatment markedly decreased LPS concentrations both in serum and feces by 35.43% and 24.51%, respectively. Furthermore, fecal acetate, propionate, and butyrate concentrations were significantly increased in SC-LCBs-treated mice compared with obese animals (*p  *<  0.05, *p  *<  0.01). These results indicate that SC-LCBs regulate secondary metabolites of gut microbiota in obese mice.

### 2.7. SC-LCBs Inhibited LPS-Dependent Pathway and Activated SCFAs-Dependent Patyway

**Figure 6:**
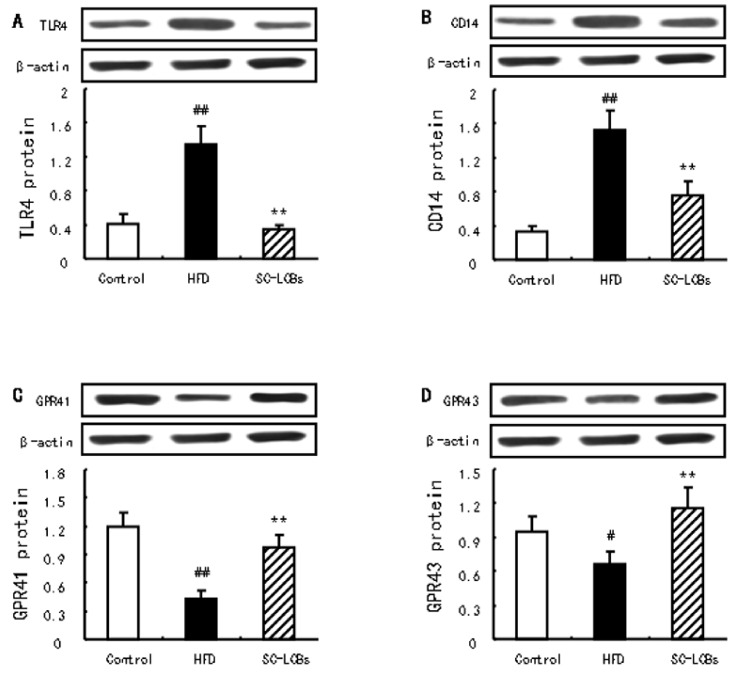
Effects of SC-LCBs on the protein expression of TLR4, CD14, GPR41, and GPR43 using Western blotting (*n* = 10). (**A**), TLR4 protein; (**B**), CD14 protein; (**C**), GPR41 protein; (**D**), GPR43 protein. ^#^ *p* < 0.05, ^##^ *p* < 0.01 compared with control mice; ** *p* < 0.01 compared with HFD mice.

The LPS-dependent TLR4/CD14 pathway is one of the pivotal signals of the development of obesity resulting from intestinal dysbacteriosis. In obese mice, the protein expression of TLR4 and CD14 was markedly elevated compared with control group (*p  *<  0.01) (Figure 6A,B). When treated with SC-LCBs, these elevations were significantly reversed in obese mice (*p  *<  0.01). In addition, SC-LCBs caused significant increases in the protein expression of GPR41 and GPR43 in HFD-fed animals (*p  *<  0.01) (Figure 6C,D), which are the crucial proteins negatively related to obesity mediated by SCFAs. These indicate that SC-LCBs can control obesity through inhibition of LPS-dependent TLR4/CD14 signal and activation of SCFAs-mediated GPRs proteins.

## 3. Discussion

Regulation of gut microbiota and its secondary metabolites has been considered as an effective approach to control obesity [25]. In the present study, we investigated the effects of SC-LCBs on antiobesity and alteration of gut microbiota. Our findings revealed that SC-LCBs significantly decreased body weight gain, adipose tissue weight and epididymal adipose cell size, serum and hepatic lipids, and hepatic lipids area, which implying the dramatic effects of SC-LCBs on anti-obesity. Microbiological analysis showed that SC-LCBs prevented the loss of beneficial gut bacteria (typically Bacteroidetes and Proteobacteria) and inhibited the increase of destructive gut bacteria (typically Firmicutes and Saccharibacteria) in HFD-fed mice. Our data also demonstrated a modulation of LPS and SCFAs production mediated by SC-LCBs, with alleviation of obesity.

Numerous studies have confirmed that there is an aberrant composition of gut microbiota in obese humans and HFD-induced obese mice to the normal individual [26,27]. We examined the relationship between the community structures of the three groups by PCA and PCoA methods on the OUTs levesl, and the data showed that there were clear separations between the three groups. Moreover, hierarchical clustering tree on OUTs levels was also showed distinct separation of the microbial composition. These suggested that SC-LCBs could play a critical role in shaping the gut microbiota community by natural selection and competing [28]. There are contradictory conclusions regarding the abundance of Bacteroidetes and Firmicutes in adipose individual. For example, Li et al. reported that high fat high fructose diet caused an increase in Firmicutes and a decrease in Bacteroidetes [29]. However, other studies showed a lower Firmicutes and higher Bacteroidetes in obese mice [30,31]. Our data showed that HFD feeding elevated Firmicutes abundance and reduced Bacteroidetes abundance, while the changes were reversed by treatment with SC-LCBs. At the genus level, SC-LCBs decreased the numbers of *unclassified_f__Ruminococcaceae*, *Ruminococcaceae_UCG-013*, *norank_f__Ruminococcaceae*, *unclassified_f__Erysipeloterichaceae*, *Ruminococcaceae_UCG-014*, and *[Ruminococcus]_torques_group*, belonging to Fimicutes, while *norank_f__Erysipelotrichaceae* increased. On the other hand, SC-LCBs increased *Parasutterella*, *Bacteroides*, *Alloprevotella*, *Rikenellaceae_RC9_gut_group*, which belong to Bacteroidetes. *Lactobacillus*, the probiotic to metabolism and negatively related to obesity [32], was significantly increased in SC-LCBs-treated mice, and the same change in order level (*Lactobacillales*). In addition, other genus bacteria, which are positively correlated with obesity, were also decreased in SC-LCBs-treated obese mice, such as *Coriobacteriaceae_UCG-002*, *Desulfovibro*, *Bifidobacterium*, *Parvibacter*, *Romboutsia*, and *Enterorhabdus* at genus level, *Clostrida*, *Bacilli*, and *Actinobacteria* at class level, and *Bifidobacteriales* and *Coribacteriales* at order level. These indicate that the direct modulating effects of SC-LCBs on gut bacteria may play an important role in the control obesity.

Dietary bioactive lipids undigested by endogenous enzymes are substrates for fermentation by *Bacteroides*, *Lactobacillus*, *Faecalibaculum*, *Lachnospiraceae*, *Alloprevotella*, *Rikenella*, *Allobaculum* etc., that produces SCFAs [33,34,35]. In the present study, the abundances of the SCFAs-producing microbiota *Faecalibaculum*, *norank_f_Bacteroidales_S24-7_group*, *Lactobacillus*, *Allbaculum*, *Bacteroides*, *Alloprevotella*, *unclassified_f__Lachnospiraceae*, *Lachnospiraceae_NK4A136_group*, *Rikenella*, and *Ruminiclostridium_9* increased in HFD-fed mice supplemented with SC-LCBs. SCFAs act as substrates or signal molecules, which are transported into blood from the intestinal lumen and subsequently taken up by body organs in the host [36]. Acetate is known to increase cholesterol synthesis, and other SCFAs can regulate lipid metabolisms [37]. This study showed that SC-LCBs remarkably increased fecal acetate, propionate, and butyrate concentrations in obese mice. Such changes may be explained by that the production and utilization of SCFAs is a consequence of the coaction of many factors, such as gut microbiota composition, body weight gain, serum and hepatic lipids, and others [38]. SCFAs-triggered the well-balanced control of opposing metabolic pathways, including lipolysis and lipogenesis, was carried out through their receptors, such as GPR41 and GPR43 lipogenesis [39]. Many papers reported that the elevations of GPR41 and GPR43 was associated with the inhibition of and obesity and its complications [40,41]. Our results showed that SC-LCBs significantly elevated the protein expression of GPR41 and GPR43, which is associated with the enhanced fecal SCFAs concentrations. These demonstrate that SC-LCBs-triggered SCFAs generation by modulation of gut bacteria positively contribute to antiobese effects by activation of GPR41 and GPR43 in HFD-fed mice.

As an endotoxin, LPS is the major component of the outer membrane of Gram-negative bacteria and can provoke obesity and its complications [42]. Significantly, the abundances of *Desulfovibrio*, *Enterorhabdus*, and *Blautia* at the genus level, belonging to Gram-negative endotoxin-producing bacteria, were reduced in response to SC-LCBs treatment in mice. These changes of gut microbiota were accompanied by decreases in LPS levels in serum and faeces. Therefore, we suggest that the inhibition of pathogenic LPS-producting bacteria by SC-LCBs might lead to a decrease of the LPS load into the systemic circulation, and might account for the antiobese effects of SC-LCBs. It is reported that LPS can impair intestinal barrier integrity [43]. In this study, SC-LCBs caused significant increases in the abundance of intestinal barrier protectors, such as *Lachnospiraceae_NK4A136_group* and *unclassified_r__Lachnospiraceae* [44]. Additionally, LPS has been repeatedly shown to positively related to obesity and especially adipocytokines [45], such as SC-LCBs-increased adiponectin, and decreased leptin, resistin, and TNF-α. In molecular, LPS-mediated TLR4/CD14 pathway has been recognized as the main mechanism linking gut microbiota and obesity [45]. In the present study, SC-LCBs inhibited TLR4 and CD14 protein expression, in conjunction with regulation of gut phyla, decrease in LPS and mitigation of body weight and fat weight. These demonstrate that SC-LCBs alleviate obesity through modulating gut microbiota and decreasing its secondary metabolite, LPS.

## 4. Materials and Methods

### 4.1. Preparation of SC-LCBs

Dried sea cucumber, *Acaudina molpadioides*, was procured from the Dinghai Marine Products Market (Zhoushan, China). SC-LCBs were extracted and analyzed as in a previous study [46]. Briefly, total lipids were obtained from the powder of sea cucumber using chloroform-methanol (2:1 *v*/*v*). Methanol containing 4 M KOH was added into the total lipids 2 h at 37 °C. The extraction was performed with chloroform-methanol-distilled water (2:1:0.9 *v*/*v*/*v*), and the chloroform layer was collected. The lipids were subsequently under HCl acidolysis 16 h at 80 °C, and then was twice extracted using *n*-hexane and diethyl ether, respectively. The crude SC-LCBs were obtained from diethylether. HPLC was performed to gain pure SC-LCBs using an Agilent 1100 HPLC system (Santa Clara, CA, USA) with a diode array detector. The yield of SC-LCBs was about 1.23% and the purity was over 95%. The molecular weights were 205.3 Da analyzed by the electrospray ionization-MS method. The components and the main chemical structure of SC-LCBs are shown in Figure S1.

### 4.2. Animal Experimental Design

Six-week-old male C57BL/6J mice (licensed ID: SCXK2014-0004) were purchased from Vital River Laboratory Animal Center (Beijing, China). Animals were housed in individual cages under a 12-hour light/dark schedule. The animals were assigned to three groups (*n* = 10 per group): Control group (fed with normal chow diet: 70% carbohydrate, 20% protein, and 10% fat), HFD group (maintained with HFD: 29% carbohydrates, 16% protein, and 55% fat), SC-LCBs group (administrated with HFD and SC-LCBs at a diet supplement dosage of 0.025%). Three groups’ animals were treated continuously for 16 weeks, and each animal was fed in metabolism cages to collect faeces. After fasted 12 h, the mice were sacrificed. All procedures were approved by the Ethics Committee of the Qingdao University.

### 4.3. OGTT

After feeding 9 weeks, OGTT experiment was performed by detecting the blood glucose levels at 0, 0.5, 1, and 2 h after intragastric administration of 2 g/kg glucose to the 5-h fasted mice. Blood glucose levels were measured using a commercial kit. The areas under curve of OGTT (AUC_OGTT_) were both calculated using Equation (1).

AUC_OGTT_ = 0.25 × A + 0.5 × B +0.75 × C + 0.5 × D (1)

where A, B, C, and D represent the blood glucose levels at 0, 0.5, 1, and 2 h, respectively.

### 4.4. Blood Glucose and Lipids Measurement

The blood from 12 h-fasting mice was used to detect fasting blood glucose levels employing a commercial kit (Jiancheng, Nanjing, Jiangsu, China). Serum was obtained with centrifugation to measure TC, TG, HDL-c, and LDL-c concentrations with commercial kits (Jiancheng), and insulin levels with an insulin ELISA kit (Invitrogen, Carlsbad, CA, USA) according to the manufacturer’s instructions.

### 4.5. Hepatic Lipids Analysis

Hepatic lipids were extracted according to the modified method of Folch et al. [47], and TG and TC levels were analyzed with the same enzymatic kits used in the serum analysis.

### 4.6. Adiokines Measurement

Obesity-related adipokine levels in serum, including adiponectin, resistin, leptin, and TNF-α, were measured using the corresponding ELISA kits (Invitrogen).

### 4.7. Hematoxylin and Eosin (H&E) Staining

The epididymal adipose and liver tissues were separated rapidly from the mice, subsequently fixed in 10% formalin, paraffin embedded, sectioned, and finally stained with H&E. Microscopic structure of the epididymal adipose and liver were observed and photographed using a fluorescence microscope (Eclipse Ci, Nikon, Tokyo, Japan). The adipose cell size and hepatic lipids area were measured by CaseViewer 2.0, and the size or area in the Control group were both defined as 1.

### 4.8. Serum and Fecal LPS Determination

Serum was diluted to 20% (*v*/*v*) with Millipore H_2_O and then heated to 70 °C to inactivate proteins. LPS levels were detected by the ELISA kit (Invitrogen). After homogenization of the faeces in ice-cold Millipore H_2_O, the supernatant was obtained under centrifugation at 7500× *g* for 15 min, and subsequently heated to 70 °C to inactivate proteins. Fecal LPS was analyzed by the aforementioned methods.

### 4.9. Fecal SCFAs Detection

Fecal SCFAs levels were evaluated according to our previous study [48]. Briefly, fecal homogenates were prepared with 1 mM 2-ethylbutyric acid in 12% formic acid, pH 2.5. After filtered with 0.22 μm polytetrafluoroethylene syringe filters, SCFAs were detected by GC/MS (5975-7890A Agilent, Santa Clara, CA, USA).

### 4.10. Fecal DNA Extraction

Fecal DNA (*n* = 5 per group) was extracted using QIAamp DNA Stool Mini Kit (Qiagen, Dusseldorf, Germany). The DNA concentration was determined by absorbance at 260 nm, and its purity was detected by the ration of A_260_ to A_280_, respectively.

### 4.11. Intestinal Microbiota Analysis

The 16S rRNA gene comprising V3 + V4 regions was amplified using a forward primer 341F (5’-CCTAYGGGRBGCASCAG-3’) and a reverse primer 806R (5’-GGACTACNNGGGTATCTTAAT-3’). PCR amplify, purify, and sequencing were all performed by Majorbio (Shanghai, China). The program of amplification was: an initial denaturation at 95 °C for 1 min, followed by 30 cycles of 98 °C for 10 s, 50 °C for 15 s, and 70 °C for 15 s, and a hold at 4 °C. The amplicons were purified and subsequently sequenced on an Illumina HiSeq platform. After splicing and filtration, sequence reads with average quality > Q20 were used for subsequent analyses. All sequences were used for the comparison of relative abundance of bacterial taxa, and were aligned into OUTs according to a 97% similarity. The taxonomic identification was performed at the Phyla, Class, Order and Genus levels. Alpha diversity was calculated using Qiime software to resolve within community abundance and diversity. Beta diversity was calculated using Weighted Unifrac Distance, PCA, and PCoA on OUTs levels for the further diversity distinction between groups.

### 4.12. Western Blotting

The epididymal adipose tissue was striped to detect LPS- and SCFAs-directived pivotal proteins using western blotting. Briefly, the proteins from epididymal adipose tissues were obtain using western lysate, and subsequently suffered with electrophoresis, transfer membrane, blocking proteins, incubation with primary antibodies, incubation with horseradish peroxidase-conjugated IgG, and chemiluminescent autography, respectively. β-Actin was considered as the internal control for the proteins of interest.

### 4.13. Statistical Analysis

For microbiota sequence data, univariate differential abundance of OTUs at the Phyla, Class, Order and Genus levels was tested by incorporating Fisher’s exact test and the false discovery rate (FDR) among control, HFD, and SC-LCBs groups and between mouse genotypes. *P* values were corrected with the Benjamini-Hochberg method to correct for the false discovery rate across multiple comparisons, which were generated using Metastats and considered significance at *p  *<  0.05. Shannon index, Simpson index, and Chao1 index were used to calculate the bacterial abundance or diversity within each sample using Student’s test in the mothur software package.

Results are expressed as mean values and standard deviations. The statistical analysis was performed with SPSS 17.0 software (SPSS Inc., Chicago, IL, USA). The difference between the control and HFD mice, between HFD and SC-LCBs groups was analyzed using Student’s test. A *p* value of < 0.05 was considered statistically significant.

## 5. Conclusions

This study demonstrated that SC-LCBs inhibited obesity in HFD-fed mice. Such beneficial effects were associated with the modulation of the community of gut microbiota. Changes in the specific bacteria by SC-LCBs could administer body weight and lipids metabolism through inhibition of LPS-depended TLR4 signaling and activation of SCFAs-depended GPRs pathway. In summary, it indicated that SC-LCBs may be used as food supplement for the control obesity and other intestinal diseases.

## Figures and Tables

**Table 1:** Effect of SC-LCBs on secondary metabolite of gut microbiota in HFD-induced obese mice ^i^.

*Parameters*	*Control*	*HFD*	*SC-LCBs*
Serum LPS (IU/mL)	0.83 ± 0.07	3.50 ± 0.45 ^##^	2.26 ± 0.32 ^*^
Fecal LPS (µg/g feces)	5.04 ± 0.70	11.3 ± 0.9 ^##^	8.53 ± 0.77 ^**^
Fecal acetate (mmol/L)	16.8 ± 1.6	7.49 ± 0.82 ^##^	12.5 ± 1.2 ^**^
Fecal propionate (mmol/L)	6.38 ± 0.67	3.50 ± 0.44 ^##^	5.07 ± 0.55 ^*^
Fecal butyrate (mmol/L)	1.46 ± 0.11	0.68 ± 0.04 ^##^	1.33 ± 0.14 ^**^

^i^: Data are presented as mean ± S.D (*n* = 10). Multiple comparisons were done using one way ANOVA. ^##^ *p* < 0.01 versus control; ^*^ *p* < 0.05, ^**^ *p* < 0.01 versus HFD.

## Supplementary Materials

The following are available online at https://www.mdpi.com/1660-3397/17/8/455/s1, Figure S1: The structures of long-chain bases form the sea cucumber, *Acaudina molpadioides.*
