# Supplementary figures and images for: Long-Chain Bases from Sea Cucumber Alleviate Obesity by Modulating Gut Microbiota
Source: Mar Drugs. 2019 Aug 1;17(8):455. doi: 10.3390/md17080455 (PMC6723202; doi:10.3390/md17080455)

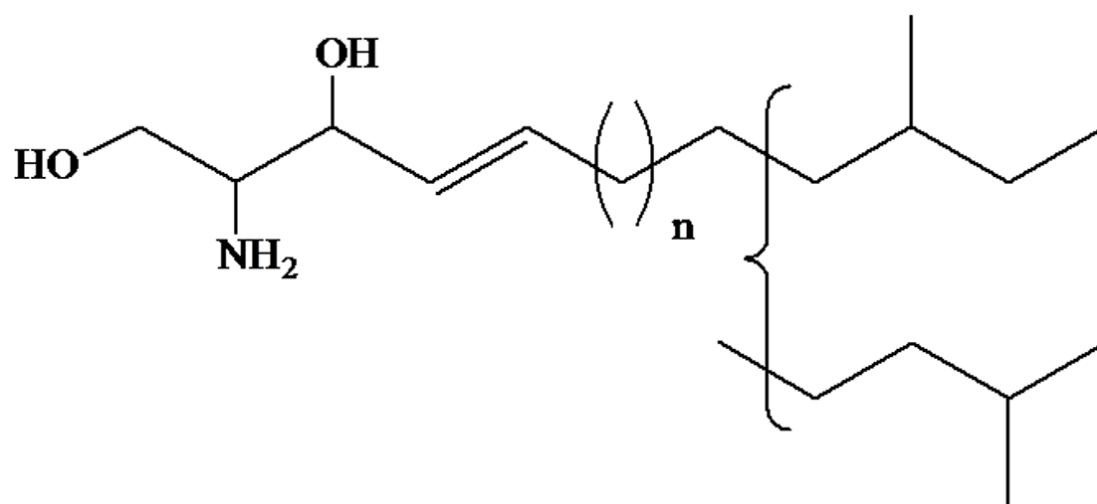

**Figure S1.** The structures of long-chain bases from the sea cucumber, *Acaudina molpadioides*.

Supplement: Supplementary file 1 [file marinedrugs-17-00455-s001.pdf]
